# Effective Data-Driven Calibration for a Galvanometric Laser Scanning System Using Binocular Stereo Vision

**DOI:** 10.3390/s18010197

**Published:** 2018-01-12

**Authors:** Junchao Tu, Liyan Zhang

**Affiliations:** College of Mechanical and Electrical Engineering, Nanjing University of Aeronautics and Astronautics, Nanjing 210016, Jiangsu, China; tujunchaochao@163.com

**Keywords:** galvanometric laser scanners, calibration, ELM, SLFN, laser projection, 3D laser measurement

## Abstract

A new solution to the problem of galvanometric laser scanning (GLS) system calibration is presented. Under the machine learning framework, we build a single-hidden layer feedforward neural network (SLFN）to represent the GLS system, which takes the digital control signal at the drives of the GLS system as input and the space vector of the corresponding outgoing laser beam as output. The training data set is obtained with the aid of a moving mechanism and a binocular stereo system. The parameters of the SLFN are efficiently solved in a closed form by using extreme learning machine (ELM). By quantitatively analyzing the regression precision with respective to the number of hidden neurons in the SLFN, we demonstrate that the proper number of hidden neurons can be safely chosen from a broad interval to guarantee good generalization performance. Compared to the traditional model-driven calibration, the proposed calibration method does not need a complex modeling process and is more accurate and stable. As the output of the network is the space vectors of the outgoing laser beams, it costs much less training time and can provide a uniform solution to both laser projection and 3D-reconstruction, in contrast with the existing data-driven calibration method which only works for the laser triangulation problem. Calibration experiment, projection experiment and 3D reconstruction experiment are respectively conducted to test the proposed method, and good results are obtained.

## 1. Introduction

A typical galvanometric laser scanner consists of two rotatable mirrors driven by two limited-rotation motors, respectively. The incoming laser beam are deflected by the mirrors, the orientations of which are uniquely determined by the control voltages applied to the two motors, so there exists a one-to-one mapping between the two input voltage signals and the outgoing laser beam.

Due to the good characteristics of high deflection speed, high positioning repeatability, low price and concise structure, galvanometric laser scanning (GLS) systems are broadly used as the key component of a variety of devices for diverse applications, such as laser marking [1,2], laser projection [3,4], optical metrology [5,6], material processing [7,8,9,10], medical imaging [11,12], etc.

These GLS-based applications can be classified into two categories: the forward applications and the backward applications. In the forward applications, e.g., laser triangulation scanning, pre-defined control voltages are input and the 3D coordinates of the laser spot of the outgoing beam hitting on the object surface need to be solved, whereas in the backward applications, e.g., laser material processing and laser marking, the position of the laser spot on the object surface is pre-defined and the control voltages need to be solved accordingly. An essential problem involved in both forward and backward applications is how to establish a system model to accurately reveal the relationship between the input and the output, which is known as system calibration.

Most existing GLS system calibration methods focus on constructing a model with a set of real structural parameters to reveal the working mechanism of the device. This kind of methods is called physical-model-based calibration. Since the GLS system does not have a single center of projection, the physical model of GLS system is relative complex and suffers from distortion. As a representative physical-model-based method, Manakov et al. [13] presented a complicated model containing up to 26 physical parameters to predict the distortions caused by installation errors. Even so, not all affecting factors are involved. In fact, system errors are difficult to be eliminated by additional parameters for a rather complex device, since too many parameters may lead to hard and non-convex optimization problems with a growing risk for local minima. To compensate the system error of the physical models, various distortion correction mechanisms were put forward [14,15,16]. These distortion correction methods are applicable only if the object that the laser beam hits on is planar [17], or only works for a limited set of outgoing rays [18]. Instead, Cui et al. [19] adapted the distortion model of a pinhole camera to represent the GLS system. This method is highly dependent on the similarities between a GLS system and a camera. Since a real GLS system does not have an optical center as that of a camera, it still needs many optimization parameters to ensure the calibration accuracy. In other words, there exists the same optimization problems as that in the physical-model-based method just mentioned.

A more reliable and flexible approach is to use the statistical learning methods (e.g., artificial neural networks, support vector machines) to approximate a model function irrespective of the specific system construction. This kind of methods establish universal regression models, which is called universal-model-based method, to describe the complex relations to be calibrated. The large amount of variables in the universal models are irrespective of specific physical meaning and are usually determined by means of supervised learning from a training data set, so they are also called data-driven methods. Along this line, Wissel et al. [20] calibrated a galvanometric triangulation device, in which a GLS system and a camera constitute a fixed triangulation structure. However, their calibration result is only applicable for the specific triangulation setup to measure the shape of 3D objects, not applicable for the backward applications mentioned above. In general, the universal-model-based methods are more adaptive to different hardware structure and can achieve higher calibration accuracy. However, this type of methods is often criticized for the low learning efficiency, because it usually requires a large amount of training data and a time-consuming iterative training procedure.

Based on the fact that each pair of voltages corresponds to only one single outgoing ray, we establish a Single Hidden Layer Feedforward Neural Network (SLFN) [21] to model the system. The SLFN directly takes the pair of voltage signals as input and the parameters of the outgoing laser beam as output. To facilitate the training data collection, a straight moving mechanism is employed. To efficiently train the established model, the Extreme Learning Machine (ELM) [22,23,24] for specially solving the SLFN is introduced. This learning method does not need to adjust the connection weights between the input layer and hidden layer in during the training. It can achieve the unique optimal solution as long as the number of hidden neuron is selected. Within the framework of ELM, we only need to solve a linear system in a closed form for completing the calibration of the SLFN model. The calibration results can be conveniently used for both the forward and the backward applications.

## 2. Materials and Methods

### 2.1. System Calibration Configuration

**Figure 1:**
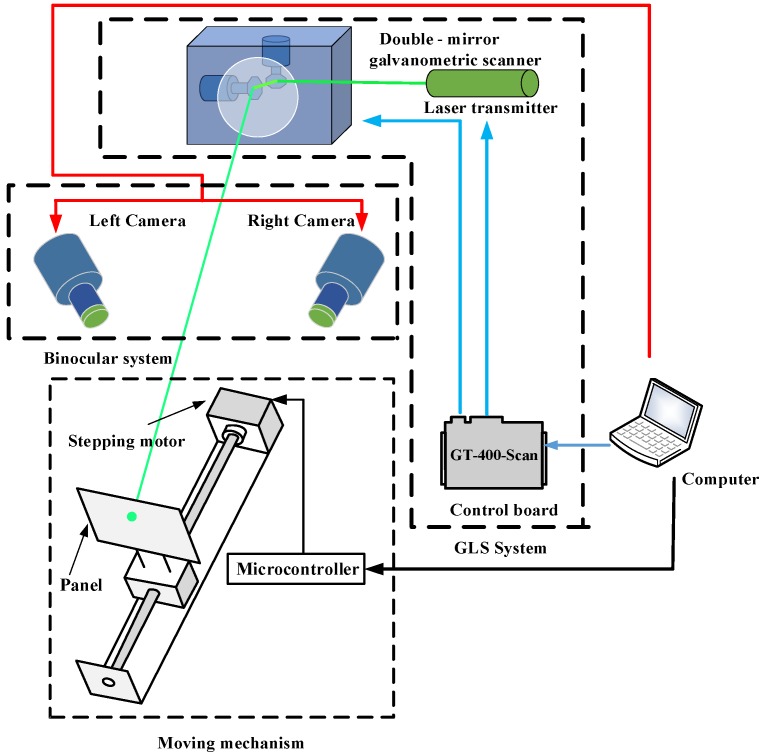
System calibration configuration.

As shown in Figure 1, the setup involved in the calibration procedure is composed of three parts: the GLS system, the binocular system and the moving mechanism. The GLS system consists of a laser transmitter, a double-mirror galvanometric scanner, a control board. The transmitter emits a laser beam, and the galvanometric scanner, including two perpendicular mirrors mounted on two separate galvanometer motors respectively, deflects the laser beam. The control board is used to control the opening or closing of the laser transmitter and the rotation angles of the dual mirrors. A smooth panel, a stepping motor, a ball screw and a microcontroller constitute the moving mechanism. The panel, coated with black flat lacquer, is fixed on the ball screw by a special clamp. The microcontroller is in charge of the stepping motor control, and the panel can do the translational motion in the viewing field of the binocular system with the help of the rotation of the ball screw driven by stepping motor. With the rapid deflection of the galvanometer, the laser beam can form a grid of laser spots on the panel. The 3D coordinates of the laser spots are obtained by the binocular system for the calibration. All the signals controlling the three parts above are sent by the same computer.

**Figure 2:**
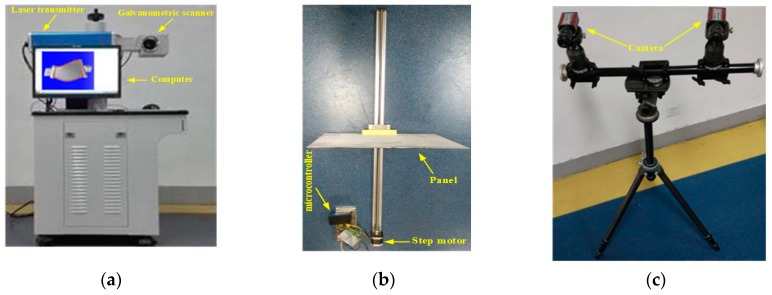
Hardware setup. (**a**) The GLS system. (**b**) The moving mechanism. (**c**) The binocular system.

Figure 2 illustrates the specific hardware used in calibration experiment. The GLS system makes use of an economic 520 nm semiconductor laser and a TSH8050A/D galvanometer (Century Sunny, Beijing, China). Both the laser transmitter and the galvanometric scanning head are controlled by a GT-400-Scan control board (GuGao, Shenzhen, China). 

The binocular system consists of two MG 419B CMOS cameras (Schneider-Kreuznach, Bad Kreuznch, Germany), two 35 mm lens and a tripod. The software for completing the whole calibration process is installed in a personal computer with 3.1 GHz and 8 GB RAM.

### 2.2. Calibration of GLS System

For the convenience of depiction, we first introduce some symbols used in this paper. We use $O_{c}-X_{c}Y_{c}Z_{c}$ to represent the camera coordinate system. Denote the digital voltage value applied to the motor of the first mirror as $d_{x}$, and that of the second mirror as $d_{y}$. The symbol V represents the two-dimensional (2D) digital control voltages ${\left[d_{x},d_{y}\right]}^{T}$. The outgoing laser beam corresponding to of a specific d is represented by l. $V={\left[v_{1},v_{2},v_{3},v_{4},v_{5},v_{6}\right]}^{T}$ represents the six-dimensional (6D) vector of l in the camera coordinate system $O_{c}-X_{c}Y_{c}Z_{c}$, where ${\left[v_{1},v_{2},v_{3}\right]}^{T}$ represents the direction of l, and ${\left[v_{4},v_{5},v_{6}\right]}^{T}$ represents a point on the beam l. The mapping between the 2D digital control voltages ${\left[d_{x},d_{y}\right]}^{T}$ and the 6D vector ${\left[v_{1},v_{2},v_{3},v_{4},v_{5},v_{6}\right]}^{T}$ is denoted as M:d→V.

#### 2.2.1. The SLFN Model

**Figure 3:**
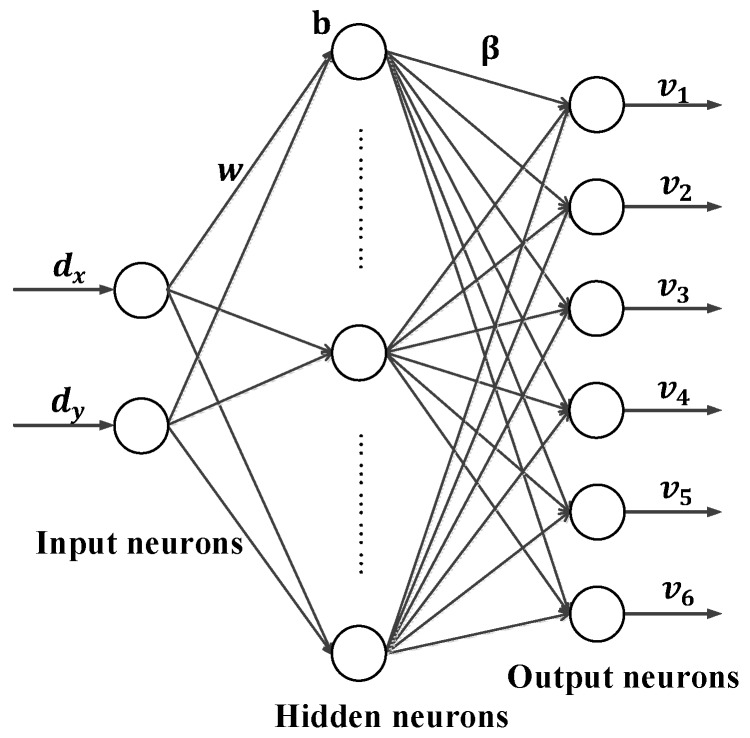
SLFN (single-hidden layer feedforward neural network) structure of the 2D to 6D mapping.

Considering the complexity of the GLS system, we treat the system modelling as a machine learning problem. More specifically, a SLFN as shown in Figure 3 is used to model the 2D-to-6D mapping relationship M:d→V. There are two neurons in the input layer, which respectively are the voltage signals $d_{x}$ and $d_{y}$. The output layer contains six neurons, which are respectively the components $v_{1}$, $v_{2}$, $v_{3}$, $v_{4}$, $v_{5}$ and $v_{6}$ of V. Denote the number of neurons in the hidden layer as L.

The model of M:d→V is formulated as:

(1) $$V=\overset{L}{\underset{j=1}{\sum }}\beta_{j}g\left(w_{j}\cdot d+b_{j}\right)$$

where $w_{j}=\left[w_{j1},w_{j2}\right]$ is the input weight vector connecting the hidden neuron and the input neurons, $\beta_{j}={\left[\beta_{j1},\beta_{j2},\beta_{j3},\beta_{j4},\beta_{j5},\beta_{j6}\right]}^{T}$ is the output weight vector connecting the hidden neuron and the output neurons, and $b_{j}$ is the bias of the j-th hidden neuron. The activation function g(x) is taken as the sigmoid function:

(2) $$g(x)=\frac{1}{1+exp(-x)}$$

After having completed the training of the SLFN model, the corresponding V to an arbitrary d can be easily obtained by Equation (1). The following two subsections describe the training method in details.

#### 2.2.2. Generating Training Data

**Figure 4:**
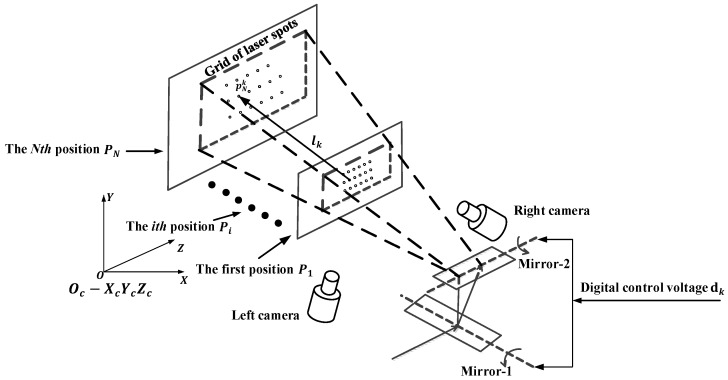
Generating the calibration data.

As shown in Figure 4, a set of control voltages $d_{k}={\left[d_{kx},d_{ky}\right]}^{T}\left(k=1,2,\cdots ,Q\right)$ is sent to control the GLS system to project a gird of laser spots onto the panel. At the same time, the panel does translational motion in the field-of-view of the binocular system, and stops at every position $P_{i},\;i=1,2,\cdots ,N$. The left camera records the image $I_{Li}$ of the laser spot grid at position $P_{i}$, and the right camera record the image $I_{Ri}$. According to the extracted image coordinate $\left(u_{Li}^{k},v_{Li}^{k}\right)$ of the laser spot $p_{i}^{k}$ in $I_{Li}$ and the coordinate $\left(u_{Ri}^{k},v_{Ri}^{k}\right)$ in $I_{Ri}$, the 3D coordinates $\left(x_{i}^{k},y_{i}^{k},z_{i}^{k}\right)$ of $p_{i}^{k}$ is computed based on the binocular stereo vision algorithm. Then the 6D vector $V_{k}={\left[v_{1},v_{2},v_{3},v_{4},v_{5},v_{6}\right]}^{T}$ of the laser beam $l_{k}$ in $O_{c}-X_{c}Y_{c}Z_{c}$ can be estimated by fitting the 3D points $\left\{p_{i}^{k},i=1,\cdots ,N\right\}$ of a common index k from the same laser beam $l_{k}$. The fitting is performed by minimizing the error measure shown in Equation (3):

(3) $$E=\overset{N}{\underset{i=1}{\sum }}{d_{i}^{k}}^{2}$$

where $d_{i}^{k}$ is the distance from the point $p_{i}^{k}$ to the laser beam $l_{k}$. Given the existence of outliers in $\left\{p_{i}^{k},i=1,\cdots ,N\right\}$, RANSAC method [25] is used to improve the fitting precision of the line. In this way, we achieve the 6D vectors $V_{k},k=1,2,\cdots ,Q$ of all the outgoing laser beams. Associating $V_{k},k=1,2,\cdots ,Q$ with the corresponding input control voltages $d_{k}={\left[d_{kx},d_{ky}\right]}^{T},k=1,2,\cdots ,Q$ the training data set ($d_{k}$, $V_{k}$), k=1,2,⋯,Q is fully achieved.

#### 2.2.3. Solving the SLFN Model

The gradient-descent-based methods are commonly used for training neural networks. These methods tune all the parameters $w_{j}$, $b_{j}$ and $\beta_{j}\left(j=1,2,\cdots ,L\right)$ of the networks iteratively in many steps. The gradient computation burden is large, since the number of the parameters is usually huge. So, these training processes are time-consuming and even may converge to local minima. To efficiently establish the 2D to 6D mapping M:d→V, we incorporate the extreme learning machine (ELM) [22,23] to solve the model formulated in Equation (1).

Given Q arbitrary samples ($d_{k}$, $V_{k}$), k=1,2,⋯,Q the SLFN with hidden neurons in Equation (1) should satisfy:

(4) $$V_{k}=\overset{L}{\underset{j=1}{\sum }}\beta_{j}g\left(w_{j}\cdot d_{k}+b_{j}\right),\;k=1,2,\cdots ,Q$$

Equation (5) can be written compactly as:

(5) $$S^{T}=H_{w,b,d}\beta$$

where:

$$H_{w,b,d}\left(w_{1},w_{2},\cdots ,w_{L},b_{1},b_{2},\cdots b_{L},d_{1},d_{2},\cdots ,d_{Q}\right)={\left[\begin{array}{ccc} g\left(w_{1}\cdot d_{1}+b_{1}\right) & \cdots & g\left(w_{L}\cdot d_{1}+b_{L}\right)\\ \vdots & \cdots & \vdots \\ g\left(w_{1}\cdot d_{Q}+b_{1}\right) & \cdots & g\left(w_{L}\cdot d_{Q}+b_{L}\right) \end{array}\right]}_{Q\times L}$$

$$\beta ={{\left[\beta_{1},\beta_{2},\cdots ,\beta_{L}\right]}^{T}}_{L\times 6},S={\left[V_{1},V_{2},\cdots V_{Q}\right]}_{6\times Q}$$

Given any small positive value ε>0 and randomly chosen $w_{j}$ and $b_{j}$, if only the activation function g(x) is infinitely differentiable, there exist L≤Q hidden nodes, such that for Q arbitrary samples $∥H_{w,b,d}\beta -S^{T}∥<\varepsilon$ holds [24]. Based on the above conclusion, we randomly assign the input weights $w_{j},j=1,2,\cdots ,L$ and the hidden layer biases $b_{j},j=1,2,\cdots ,L$. Then the hidden layer output matrix $H_{w,b,d}$ in Equation (6) is fully determined, and the model in Equation (1) can be simply considered as a linear system. The output weights in β is determined by:

(6) $$\beta =H_{w,b,d}^{+}S^{T}$$

where $H_{w,b,d}^{+}$ is the generalized inverse matrix of $H_{w,b,d}$. This is the smallest norm least square solution to the linear system. The solved β, together with the randomly chosen $w_{j}$ and $b_{j}$, completely determine the model in Equation (1).

### 2.3. Validations

In order to verify the accuracy and efficiency of the proposed calibration method, cross validation experiment and target shooting experiment are performed respectively. Moreover, we provide both the forward and the backward applications based on the calibrated mapping M:d→V. The achievement of these applications fully testifies the wide applicability of the proposed method in various applications.

#### 2.3.1. Cross Validation

**Figure 5:**
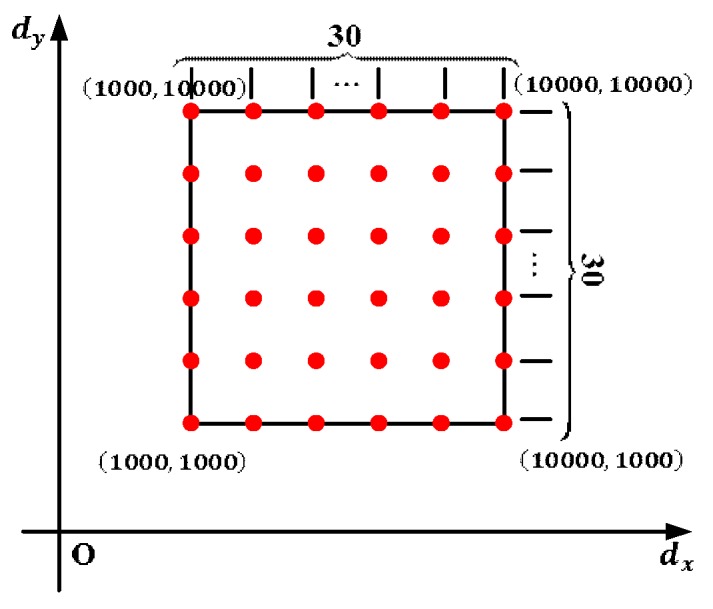
Distribution of the 900 digital voltages $d_{k}={\left[d_{kx},d_{ky}\right]}^{T}\left(k=1,2,\cdots ,900\right)$.

Nine hundred (900) pairs of digital voltages $d_{k}={\left[d_{kx},d_{ky}\right]}^{T}\left(k=1,2,\cdots ,900\right)$ are input to generate 900 outgoing laser beams in the field-of-view of the binocular system. The 900 pairs of digital voltages are uniformly spaced on the virtual digital plane as shown in Figure 5. According to the method in Section 2.2.2, the sample data set ($d_{k}$, $V_{k}$) (k=1,2,⋯,900) is obtained. Here the number of the position N is set as 100. The distance between the first position $P_{1}$ and the last position $P_{N}$ is about 1 m, and the first position $P_{1}$ is approximately 2 m away from the GLS system. With the help of the moving mechanism, the whole sampling procedure costs less than 10 min.

To evaluate the performance of the new calibration method, the 10-fold cross validation is adopted. We randomly draw the one-tenth of the sample data as the test data, and use the rest of the sample data to train the SLFN model by means of the method in Section 2.2.3. For arbitrary input voltages $d_{j}^{tst}={\left[d_{jx}^{tst},d_{jy}^{tst}\right]}^{T},j=1,2,\cdots ,90$ in the test data set, its corresponding outgoing beam $l_{j}^{tst}$ is represented by the 6D vector $V_{j}^{tst}={\left[v_{j1}^{tst},v_{j2}^{tst},v_{j3}^{tst},v_{j4}^{tst},v_{j5}^{tst},v_{j6}^{tst}\right]}^{T}$, which is fitted out with the process in Section 2.2.2.

Meanwhile, we calculate a 6D vector denoted as $V_{j}^{M}={\left[v_{j1}^{M},v_{j2}^{M},v_{j3}^{M},v_{j4}^{M},v_{j5}^{M},v_{j6}^{M}\right]}^{T}$ for every $d_{j}^{tst}$ through the established mapping M:d→V. If the error of the established mapping M:d→V tends to be zero, the beam $l_{j}^{tst}$ and the beam $l_{j}^{M}$ determined by $V_{j}^{M}$ should be the same line. However, the evaluation of the difference between $l_{j}^{tst}$ and $l_{j}^{M}$ is difficult. So, an equivalent evaluation method is introduced here.

The corresponding input digital voltages $d_{j}^{M}={\left[d_{jx}^{M},d_{jy}^{M}\right]}^{T}$ of the spatial beam $l_{j}^{M}$ is given by:

(7) $$d_{j}^{M}=\arg\min_{d}D_{j}\left(d\right)$$

where $D_{j}\left(d\right)$ is the distance from the point $p_{j}^{tst}={\left[v_{j4}^{tst},v_{j5}^{tst},v_{j6}^{tst}\right]}^{T}$ to the corresponding laser beam of the digital voltages $d={\left[d_{x},d_{y}\right]}^{T}$, and $p_{j}^{\mathrm{tst}}$ is a point on the beam $l_{j}^{tst}$. The difference between $d_{j}^{M}$ and $d_{j}^{tst}$ is denoted as $\varepsilon_{j}$ and defined by Equation (8). In the ideal situation that $l_{j}^{tst}$ and $l_{j}^{M}$ exactly coincide, $\varepsilon_{j}$ is a zero vector:

(8) $$\varepsilon_{j}=\left(\begin{array}{c} \varepsilon_{jx}\\ \varepsilon_{jy} \end{array}\right)=\left(\begin{array}{c} d_{jx}^{tst}-d_{jx}^{M}\\ d_{jy}^{tst}-d_{jy}^{M} \end{array}\right)$$

Then we use the root mean squared error (RMSE) measure in Equation (9) to estimate the error of the calibrated mapping M:d→V. Obviously, the smaller the value of $E_{d}$ is, the higher the accuracy of the calibration is:

(9) $$E_{d}=\sqrt{\overset{Q^{tst}}{\underset{j=1}{\sum }}\left({\left(d_{jx}^{tst}-d_{jx}^{M}\right)}^{2}+{\left(d_{jy}^{tst}-d_{jy}^{M}\right)}^{2}\right)/Q^{tst}}$$

where $Q^{tst}$ is the number of the test samples.

In the process of solving the SLFN model by the method mentioned in Section 2.2.3, an inevitable problem is the determination of the number of hidden neurons L in generalized SLFN. To investigate the influence of the hidden neuron number L on the calibration accuracy, we also tested the variation tendency of regression error $E_{d}$ with respect to the equal increase of L. Considering the possible influence of the training sample number Q on the variation tendency, three groups of test experiments with different number of training samples (*Q* = 810,405,270) are respectively conducted.

#### 2.3.2. Target Shooting

**Figure 6:**
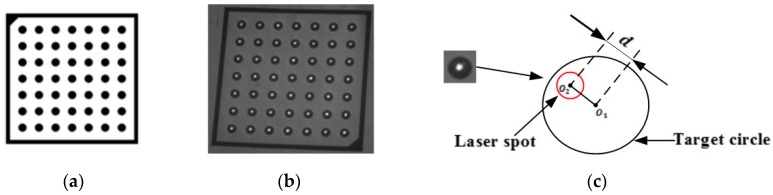
Design of target shooting experiment. (**a**) The pattern of target circles. (**b**) Experiment result of target shooting. (**c**) The distance between the centers of laser spot and target circle.

To further verify the proposed calibration method, we design a pattern of 49 target circles (shown in Figure 6a) to be shot by the laser beam of the calibrated GLS system. The pattern is placed in the field-of-view of the binocular system, and the 3D coordinates ${\left[x_{n}^{dst},y_{n}^{dst},z_{n}^{dst}\right]}^{T},\;n=1,2,\cdots ,49$ of the circle centers $p_{n}^{dst},n=1,2,\cdots 49$ are obtained by the binocular system. By using the calibrated mapping M:d→V and the coordinates $p_{n}^{dst},n=1,2,\cdots ,49$, the digital voltages $d_{n}^{dst}={\left[d_{nx}^{dst},d_{ny}^{dst}\right]}^{T},\;n=1,2,\cdots ,49$ are achieved by:

(10) $$d_{n}^{dst}=\arg\min_{d}D_{n}\left(d\right)$$

where $D_{n}\left(d\right)$ is the distance from the 3D point $p_{n}^{dst}={\left[p_{nx}^{dst},p_{ny}^{dst},p_{nz}^{dst}\right]}^{T}$ to the spatial beam $l^{d}$, which is the corresponding laser beam of the digital voltages $d={\left[d_{x},d_{y}\right]}^{T}$. Utilizing these digital voltages, we control the GLS system to shoot the target circles as shown in Figure 6b. The coordinates of the laser spot centers shot on the pattern $p_{n}^{spot},n=1,2,\cdots ,49$ are also obtained by the binocular system. Then we use the standard deviation of the distance $S_{d}=\sqrt{\frac{1}{49}\sum_{n}{\left(p_{n}^{dst}-p_{n}^{spot}\right)}^{2}}$ to measure the shooting precision as shown in Figure 6c.

#### 2.3.3. 3D Reconstruction

3D reconstruction is the representative of forward applications of GLS system. In this application, pre-defined input digital voltages are known and the 3D coordinates of the laser spots hitting on the object need to be solved. More specifically, digital voltages $d_{m}^{dst}={\left[d_{mx}^{dst},d_{my}^{dst}\right]}^{T}$, $m=1,2,\cdots ,Q_{m}$ ($Q_{m}$ is the number of the digital signals) are input to control the GLS system to project $Q_{m}$ laser spots on the surface of an object to be reconstructed in 3D space, then the image of the laser spots is taken by the left camera. Using the extracted pixel coordinates ${\left[u_{m}^{dst},v_{m}^{dst}\right]}^{T}$ of the laser spots and the intrinsic parameters of the left camera, we get a set of straight lines $l_{m}^{Cam}$ through the origin of the camera coordinate system by Equation (11):

(11) $$V_{m}^{Cam}=A^{-1}\left(\begin{array}{c} u_{m}^{dst}\\ v_{m}^{dst}\\ 1 \end{array}\right),m=1,2,\cdots ,Q_{m}$$

where A is the intrinsic parameter matrix of the left camera, and $V_{m}^{Cam}$ is the direction vector of $l_{m}^{Cam}$. The vector $V_{m}^{GLS}$ of the laser beam $l_{m}^{GLS}$ going through the spot is easily obtained by substituting the input digital voltages $d_{m}^{dst}$ into the calibrated mapping M:d→V. We then make use of the coordinate ${\left[x_{m}^{dst},y_{m}^{dst},z_{m}^{dst}\right]}^{T}$ of the midpoint $p_{m}^{dst}$ of the common perpendicular between $l_{m}^{GLS}$ and $l_{m}^{Cam}$ to represent the intersection between the two beams, i.e., the 3D coordinates of the laser spot. In this way, we get a number of coordinates ${\left[x_{m}^{dst},y_{m}^{dst},z_{m}^{dst}\right]}^{T},\;m=1,2,\cdots ,Q_{m}$ of all the laser spots on the surface.

In the real 3D Reconstruction experiment, we choose the surface of an engine blade as reconstructed object since it is a free-form surface. In addition, 3D reconstruction of the engine blade by the date-driven triangulation method [20] is also implemented for comparison. To evaluate the reconstruction accuracy, we use the commercial ATOS system (GOM, Brunswick, Germany) to measure the surface in advance. The measurement accuracy of the ATOS system is high (0.03 mm), so we approximately take the measuring result of ATOS system as the real value for comparison. By the iterative closest point (ICP) method [26], we can achieve the registration result between the reconstructed 3D points and the measuring result of ATOS system. Then we calculate the root mean square error (RMSE) by Equation (12) to measure the reconstruction error:

(12) $$\mathrm{RMSE}=\sqrt{\overset{Q_{m}}{\underset{m=1}{\sum }}\varepsilon_{m}^{2}/Q_{m}},\;m=1,2,\cdots ,Q_{m}$$

where $\varepsilon_{m}$ represents the distance between the 3D point $p_{m},\;m=1,2,\cdots ,Q_{m}$ and the closest point belong to the data cloud measured by ATOS system after the ICP registration.

#### 2.3.4. Projection Positioning

Laser projection positioning is the representative of backward applications of GLS system. In this application, the position of the laser spot on the 3D object is pre-defined and the digital control voltages need to be solved accordingly. In this validation, a serial of laser spots needs to be projected on the edge contour of an object by the calibrated GLS system. The 3D coordinates of the visual feature points placed on the object and the CAD model of the object in the object coordinate system are measured in advance. By the visual feature points, the coordinates ${\left[x_{e}^{tgt},y_{e}^{tgt},z_{e}^{tgt}\right]}^{T},e=1,2,\cdots ,Q_{e}$ ($Q_{e}$ is the number of the discrete points). $p_{e}^{tgt},e=1,2,\cdots ,Q_{e}$ on the edge contour in the camera coordinate system are achieved. Utilizing $p_{e}^{tgt}={\left[x_{e}^{tgt},y_{e}^{tgt},z_{e}^{tgt}\right]}^{T},e=1,2,\cdots ,Q_{e}$ and the calibrated mapping M:d→V, the digital voltages $d_{e}^{tgt}={\left[d_{ex}^{tgt},d_{ey}^{tgt}\right]}^{T},\;e=1,2,\cdots ,Q_{e}$ are obtained by:

(13) $$d_{e}^{tgt}=\arg\min_{d}D_{e}\left(d\right)$$

where $D_{e}\left(d\right)$ is the distance from the 3D point $p_{e}^{tgt}={\left[x_{e}^{tgt},y_{e}^{tgt},z_{e}^{tgt}\right]}^{T}$ to the corresponding laser beam of the digital voltages $d={\left[d_{x},d_{y}\right]}^{T}$. We then send the digital voltages $d_{e}^{tgt},\;e=1,2,\cdots ,Q_{e}$ to the GLS system to realize the laser projection positioning of the edge contour of the object.

In the real projection positioning experiment, we choose the edge of an engine blade as the target to be projected since the edge is the 3D free curve. The CAD contour of the edge is discretized into 131 points, and 3D coordinates of these discrete points in the binocular coordinate system are obtained by the feature points placed on the surface of the engine blade. The engine blade is about 2.5 m away from the GLS system.

## 3. Results

The results of the laboratorial experiments and the accuracy test for Cross Validation, Target Shooting, 3D Reconstruction and Projection Positioning are respectively illustrated in this Section.

### 3.1. Cross Validation Experiment

**Figure 7:**
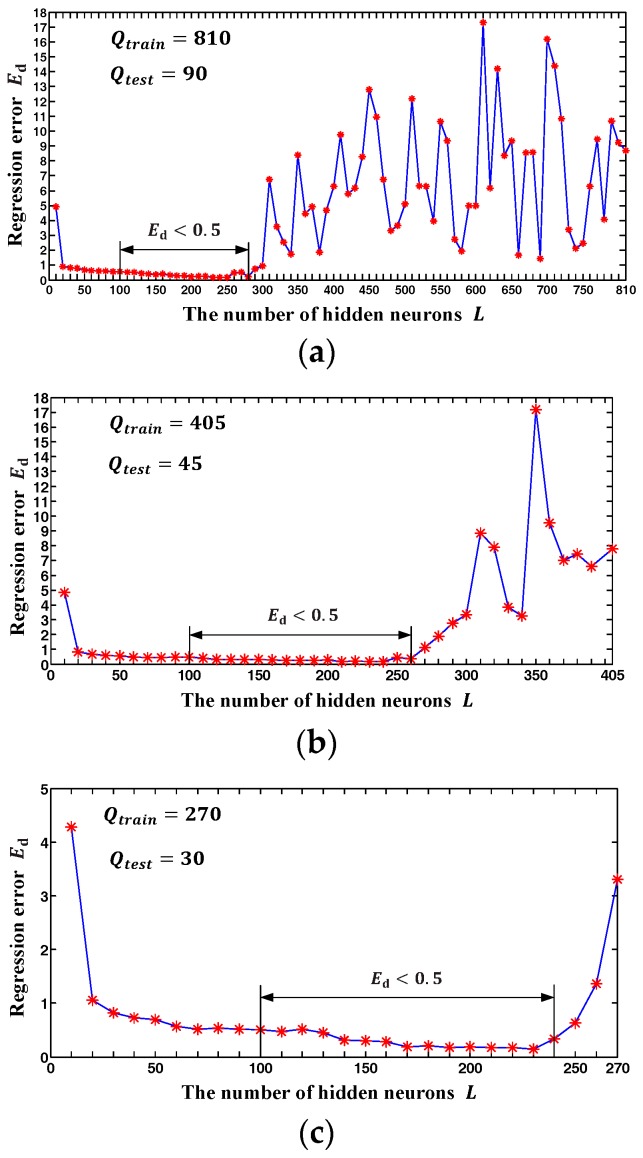
The variation tendency of regression error $E_{d}$ with respect to the equal increase of L. (**a**) The number of sample is 900. (**b**) The number of sample is 450. (**c**) The number of sample is 300.

The test results of the influence of the hidden neuron number L on the calibration accuracy are shown in Figure 7. According to the theorem proved by Huang et al. [21,22], the training error is zero when the hidden neuron number L is equal to the training sample number Q. However, it cannot ensure good generalization performance of the established SLFN, i.e., it cannot guarantee to achieve low regression error $E_{d}$. Figure 7a,b shows that the regression error $E_{d}$ rapidly increases when L exceeds 300, which indicates that the established SLFN model can be seriously over fitted by improperly increasing the hidden neuron number L. On the other hand, insufficient hidden neuron number may lead to an under fitting problem. As shown in Figure 7a–c, the value of $E_{d}$ is also relatively high when L is under 20. Fortunately, all the values of $E_{d}$ for L ∈ [100, 200] are less than 0.5 as shown in Figure 7a–c. This can be explained by the fact that the model complexity involved in a specific problem is constant and should not change with the training sample number. This can be explained by the fact that the model complexity involved in a specific problem is constant and should not change with the training sample number. The hidden neuron number L in the SLFN substantially determines the complexity of the established model of the GLS system. Based on the experiment results and the above explanation, we conclude that *L* ∈ [100, 200] and Q>L can achieve a good generalization performance for the GLS system calibration problem. Therefore, we choose the L=150∈[100, 200] as the hidden neuron number of the established SLFN in the follow-up experiments.

**Figure 8:**
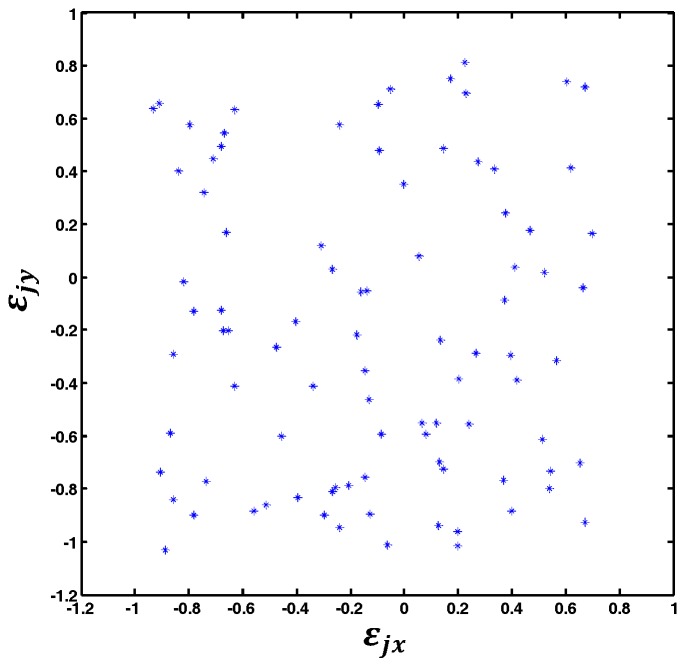
Error distribution of the input digital voltages.

Once L is selected, the structure of the SLFN model of the GLS system is determined. With the determined SLFN and the training data set ($d_{k}^{train}$, $V_{k}^{train}$) (k=1,2,⋯,810), the mapping M:d→V of GLS system is finally determined. When M:d→V is calibrated, the actual distribution of $\varepsilon_{j}=\left(\varepsilon_{jx},\varepsilon_{jy}\right),j=1,2,\cdots ,90$ is shown in Figure 8. The actual distribution of $\left(\varepsilon_{jx},\varepsilon_{jy}\right),j=1,2,\cdots ,90$ is shown in Figure 8. According to the XY2-100 protocol of the GLSs we used, the voltage signals $d={\left[d_{x},d_{y}\right]}^{T}$ are dimensionless quantities. The range of $d_{x}$ and $d_{y}$ is −32768 to 32767 respectively, and the rotation angle range of the mirrors is −12.5° to 12.5° respectively. As shown in Figure 8, the maximums of $\varepsilon_{jx}$ and $\varepsilon_{jy}$ (j=1,2,⋯,90) are −0.957 and −1.04 respectively which can lead to 6.37 μrad and 6.91 μrad rotation error for the two GLS mirrors respectively.

**Figure 9:**
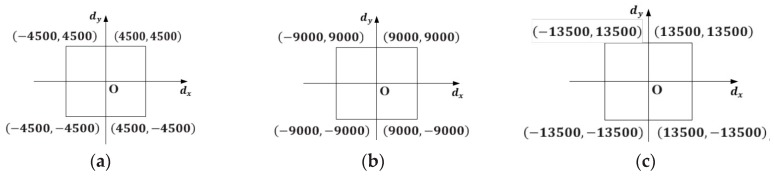
Virtual digital planes of different size corresponding to the three different sampling densities. (**a**) The sampling interval is 300. (**b**) The sampling interval is 600. (**c**) The sampling interval is 900.

To investigate the association between the sampling density and the calibration accuracy, 900 pairs of digital voltages $d_{k}={\left[d_{kx},d_{ky}\right]}^{T},k=1,2,\cdots ,900$ are uniformly spaced on three virtual digital planes (Figure 9) of different sizes. The physical-model-based method [13] and the Look-Up-Table (LUT) based method are also realized for comparison. The physical-model-based calibration constructs a model with a set of real structural parameters and solves these parameters by a Levenberg-Marquardt [27] optimization. Utilizing the training data set, the LUT-based calibration can determine the other space vectors of laser beams corresponding to the signals within the sampling range by a linear interpolation. Whatever method is used, the goal of the GLS system calibration is to determine the space vector of the laser beam corresponding to an arbitrary control signal. Therefore, we can use the same data set ($d_{k}$, $V_{k}$) (k=1,2,⋯,900) achieved in Section 2.3.1 for the three different calibration methods. Calibration results with different sampling densities are listed in Table 1.

As shown in Table 1, the accuracy of the physical-model-driven method is the lowest compared to the LUT method and the proposed method, and the LUT method is remarkably inefficient among the three calibrations. Both the accuracy and the running time of the proposed method have obvious comparative advantages.

**Remark.**  The running time of the physical-model-based method is the time for solving all the parameters of the model by an optimization. The running time of the LUT-based method is the time for establishing the LUT by a linear interpolation. The running time of the proposed method is the time for solving the SLFN model with the training data (including the time for linear fitting mentioned in Section 2.2.2).

### 3.2. Target Shooting Experiment

**Figure 10:**
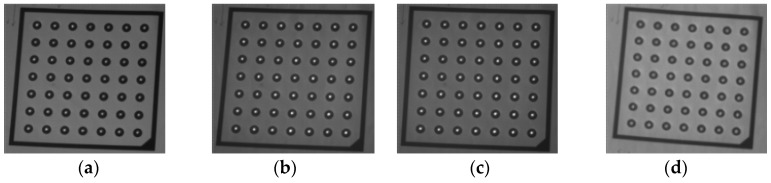
Shooting experiment results at four different distances from the GLS system. (**a**) 2 m. (**b**) 2.35 m. (**c**) 2.7 m. (**d**) 3 m.

**Figure 11:**
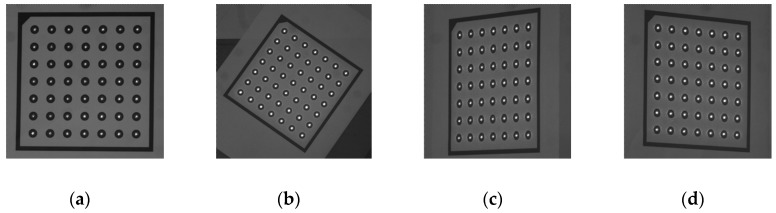
Shooting experiment results with four different poses (**a**–**d**) at a same distance (2.5 m) from the GLS system.

Two groups of experiment are conducted. The first group is focused on the influence of the shooting distance on the shooting accuracy. The experiment results at four different shooting distances are shown in Figure 10. The second group is focused on the influence of the target poses. The experiment results are shown in Figure 11 with four target poses at the same distance (2.5 m). The standard deviations $S_{d}$ for different shooting distances are listed in the first four rows of Table 2. The last four rows in Table 2 list $S_{d}$ for different target poses at 2.5 m distance.

As listed in Table 2, the error of target shooting is less than 0.346 mm within the range of 1 m from the first sampling position $P_{1}$ to the last position $P_{100}$. The average shooting error of the 8 experiments is 0.28 mm.

### 3.3. 3D Reconstruction Experiment

**Figure 12:**
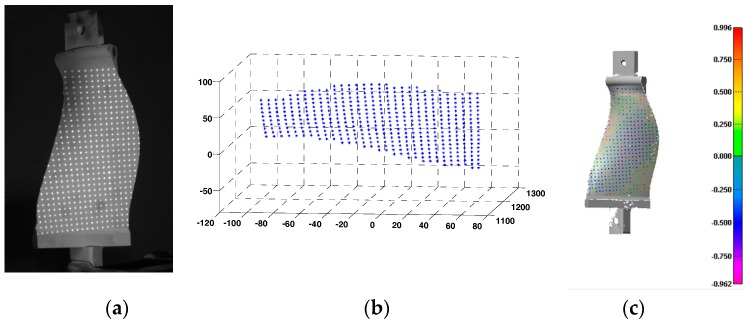
3D reconstruction of an engine blade by the proposed method. (**a**) Laser spots are projected onto the surface of the engine blade to be reconstructed. (**b**) Reconstructed 3D points. (**c**) Error distribution of the registration.

According to the reconstruction method mentioned in Section 2.3.3, a number of laser spots are projected onto the surface of an engine blade as shown in Figure 12. The reconstructed 3D points of the surface are achieved in Figure 12b. The registration result between the reconstructed 3D points and the measuring result of ATOS system is shown in Figure 12c. Although the error distribution of the registration shows the maximum error is 0.996 mm, most errors of the reconstructed points are in this interval [−0.5, 0.5] as shown in Figure 12c. A few points of relatively large error are distributed in the surface edge where the laser spot imaging is difficult.

In the date-driven triangulation method, it also needs to train a data-driven model for direct triangulation. The input features of the model built by this method consist of 2D coordinates of laser spots in camera image and the control voltage at the drives of both galvanometric mirrors. The output feature of the model is the 3D coordinate of the laser spot. Therefore, we can directly use the experimental data (image coordinates $\left(u_{Li}^{k},v_{Li}^{k}\right)$ and the 3D coordinates $\left(x_{i}^{k},y_{i}^{k},z_{i}^{k}\right)$) obtained in Section 2.2.2 to train the triangulation model. The respective reconstruction results are shown in Table 3.

We can find in Table 3 that the reconstruction accuracy of the two different methods is closed. However the efficiency of the proposed method is higher than the data-driven triangulation (Table 3). It should be noted that we also adopt the linear system built by the method mentioned in Section 2.2.3 to solve the triangulation model. If the other nonlinear methods (like the Support Vector Machine (SVM), the Gaussian Processes (GPs)) are used to solve this problem, it will lead to explosive time growth for GLS system calibration. As mentioned by Wissel et al. [20], the training time by SVM is 8.91 min, and the time by GPs is more than 20 h. Moreover, the number of the training sample in their experiments (7193 points) is less than one tenth of ours (90,000 points). As we all know, the training time of the model is proportional to the number of the training sample.

### 3.4. Projection Positioning Experiment

**Figure 13:**
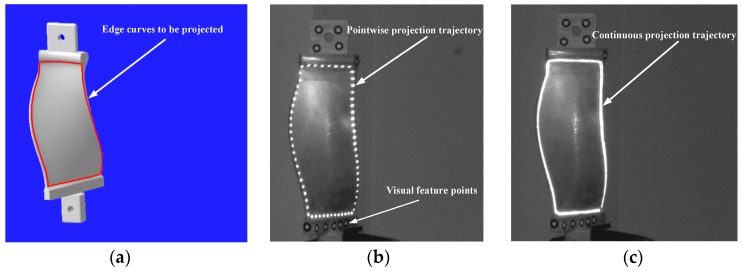
The projection positioning experiment. (**a**) The CAD model of the engine blade. (**b**) Projection result in point scan mode. (**c**) Projection result in linear interpolation scan mode.

The CAD model of the engine blade is shown as Figure 13a, and the red curve is the target contour. The GLS system has two scan modes: point scan and linear interpolation scan. In the point scan mode, the laser transmitter is turned off between inputting any two adjacent pairs of digital signals, and the laser trajectory is pointwise as shown in Figure 13b. Whereas in the linear interpolation scan mode, the laser transmitter keeps turned on all the time and the laser trajectory are continuous as illustrated in Figure 13c.

**Figure 14:**
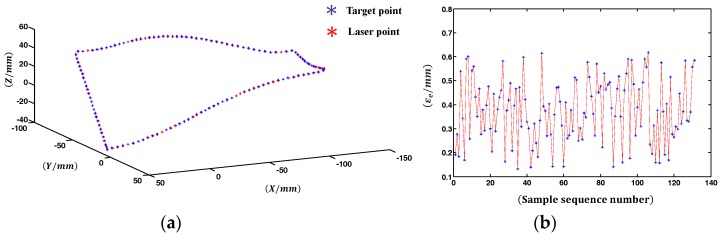
Evaluation of the projection accuracy. (**a**) The theoretical target point (in blue) and the actual laser projection point (in red). (**b**) Projection errors of the 131 discrete points.

To quantitatively evaluate the laser projection accuracy, the 3D coordinates of the 131 laser spots actually projected on the engine blade, denoted as $p_{e}^{tgt},\;e=1,2,\cdots ,131$ are obtained by using the binocular system. The theoretical target contour and the actual laser projection contour (i.e., the linear interpolation of the points $p_{e}^{tgt},\;e=1,2,\cdots ,131$) are shown in Figure 14a. The Euclidean distance $\varepsilon_{e}$ between $p_{e}^{tgt}$ and $p_{e}^{tgt},\;e=1,2,\cdots ,131$ are calculated and illustrated in Figure 14b. The average projection positioning error is $\bar{\varepsilon }=\sum_{e=1}^{131}\varepsilon_{e}/131$ = 0.32 mm, and the maximum of $\varepsilon_{e},\;e=1,2,\cdots ,131$ is 0.62 mm, the standard deviation $\sigma =\sqrt{\sum_{e}{\left(\varepsilon_{e}-\bar{\varepsilon }\right)}^{2}/131}=0.28\;\mathrm{mm}$.

## 4. Discussion

In the solution of the SLFN model of the GLS system, we determine the number of hidden neurons L based on the investment in Section 3.1. The value of L is also suitable for other GLS systems or different sampling date set since L represents the system complexity that is constant. The SLFN determined by L is equivalent to the physical model of the GLS system built in physical-model-based calibration, and the parameter setting of the physical model is not changed for a GLS system whose inner structure is fixed. According to the actual distribution of $\varepsilon_{j}=\left(\varepsilon_{jx},\varepsilon_{jy}\right)$ in Section 3.1, we consider the established SLFN model has good imitation ability on the simulation of the real GLS system. In addition, we indirectly test the calibration accuracy at different positions of the sampling region by the target shooting experiment. The good accuracy performance of shooting indicates the consistency of the calibration result in the whole sampling range (1 m) is also great. This accuracy consistency allows the follow-up GLS-based applications to be less affected with the size or the position of the experimental object.

The contrast experiment with the other two calibration methods of GLS system is also conducted, and the results are shown in Table 1. The main reason for the poor performance of the physical-model-driven method is that the physical model of the GLS system cannot involve some affecting factors, such as the nonlinear relation between the mirror rotation angles and the applied digital voltages. Furthermore, the calibration results is wrong sometimes since it optimizes too many parameters and strongly depends on the given initial values of the parameters. Both the LUT method and the proposed method have a better performance when sampling in the virtual digital plane shown in Figure 9a, but the accuracy of the LUT method declines faster than the proposed method as the sample density reduces. This phenomenon of precision falling reflect a fact that the accuracy of the LUT-based calibration is more affected by the sampling density than other calibrations with a model. Substantially, the accuracy of a location in the constructed LUT mainly depends on the neighborhood sampling points since the calibration outcome of the location is achieved by neighborhood point interpolation. Relatively, the accuracy of the calibration with a model is the combination of all the sample data. On top of that, constructing a high resolution lookup table shows higher computational cost (Table 1). The reason lies in that the space vectors corresponding to the non-sampled digital voltages in a certain digital area needs to be calculated by interpolation. As the virtual digital plane (Figure 9) grows in size, the computational burden of interpolation significantly increases, which results in the ever-increasing time consumption (Table 1). As mentioned in Section 2.2.3, the model in Equation (1) is a linear system, so the proposed method is much more efficient (only 0.872 s). The shorter the training time is, the quicker the whole calibration process is. The rapid calibration brings great convenience to various GLS-based field applications.

In the 3D reconstruction experiment, although the reconstruction accuracy of the proposed method is closed to that of the data-driven triangulation method, the proposed method has two obvious advantages. On the one hand, the training time of our method (0.872 s) is much less than that of the data-driven triangulation method (8.16 s). When the sampling density and sampling region of the two methods are the same, fitting the laser spots that belong to the same laser beam into a straight line (the 6D vector) in our method can greatly decrease the number of the training data. Less input training data means the less number of equations in Equation (6), which can remarkably reduce the computation time for solving the equations. The linear fitting itself in our method costs 0.801 s in this experiment, which has been involved in the total training time (0.872 s). On the other hand, the calibration result M:d→V of our method is the relation between the input digital voltages and the corresponding outgoing laser beam, which is independent of the binocular system. So, it is applicable to both forward and backward applications. However, the calibrated mapping $M:\left(d_{x}^{dst},d_{y}^{dst},u^{dst},v^{dst}\right)\to \left(x^{dst},y^{dst},z^{dst}\right)$ of the data-driven triangulation method [20] contains the 2D image coordinates $\left(u^{dst},v^{dst}\right)$ of the laser spot in its 4D input, leading to the inapplicability for the backward applications.

The proposed calibration also applies to the projection positioning of 3D contour, which is widely used in prepreg layup of composite. Compared with the accuracy requirement of aeronautic composite layer (±0.7 mm) [28], the projection errors (Figure 14b) indicates that the accuracy of the projection positioning experiment satisfies the requirement. Similar to the principle of target shooting, this application changes the target point into the discrete point of 3D contour to be projected. Therefore, without consideration to the machining precision of the impeller to be projected, the target shooting accuracy determines the projection positioning accuracy. The standard deviation σ calculated in Section 3.4, representative of the positioning precision, is in line with the expected accuracy range.

Besides the calibration method, the sampling data for training the system model is another factor to determine the calibration precision. In other words, the accuracy of the space vector $V_{k}$ corresponding to the digital $d_{k}$ is important for a good calibration result. For achieving good space line fitting results, we usually need to collect the 3D sample points in a relative long distance (1 m in this paper). However the depth of the binocular vision system is limited. Inevitably, the accuracy of the 3D sample points outside of the depth is affected due to the degradation of the sampling image quality. Therefore, we can take some methods (such as the image deblurring) to improve the accuracy of the sampling data in the future.

## 5. Conclusions

An accurate and efficient method for calibrating the GLS system is proposed. Based on the one-to-one mapping between the input digital voltages d and the space vector of the outgoing laser beam V, we establish the system model M:d→V using a single-hidden layer feedforward neural network (SLFN). Within the extreme learning machine framework, the system model calibration is completed by only solving a linear system, which avoids the long training time required by most machine learning methods. More importantly, taking the space vector of the outgoing laser beam V as the output of the established SLFN greatly reduces the number of equations in the linear system, thereby further improves the computational efficiency. Calibration experiments demonstrate that the proposed method outperforms the mainstream approaches in accuracy and efficiency. In addition, the calibrated mapping M:d→V allows the calibration method to handle various GLS-based applications. The 3D reconstruction and the laser projection positioning experiments validate the versatility of our method.

## Figures and Tables

**Table 1:** Performance of the three calibration methods with different sampling densities. (**a**), (**b**) and (**c**) respectively represent the different sample density shown in Figure 9.

	Physical-Model	LUT	The Proposed Method
$E_{d}$(a)	2.82	0.65	0.56
$E_{d}$(b)	3.09	1.46	0.67
$E_{d}$(c)	3.36	2.98	0.82
Running time (a)	61.2s	126s	0.872s
Running time (b)	61.2s	508s	0.872s
Running time (c)	61.2s	1188s	0.872s

**Table 2:** Performance measure $S_{d}$ at different shooting distances. (**a**), (**b**), (**c**) and (d) respectively represent the four different poses of the target in 2.5 m.

Shooting Distance (m)	$S_{d}$ (mm)
2	0.324
2.35	0.282
2.7	0.278
3	0.346
2.5(a)	0.242
2.5(b)	0.244
2.5(c)	0.256
2.5(d)	0.269

**Table 3:** Reconstruction of an engine blade by two different methods.

Calibration Methods	RMSE of Reconstruction (mm)	Time for Training a Model (s)
The proposed method	0.462	0.872
The data-driven triangulation	0.554	8.16
